# Draft Genome Sequence of *Synechococcus* sp. Strain CB0101, Isolated From the Chesapeake Bay Estuary

**DOI:** 10.1128/genomeA.01111-13

**Published:** 2014-01-09

**Authors:** David Marsan, K. Eric Wommack, Jacques Ravel, Feng Chen

**Affiliations:** The Institute of Marine and Environmental Technology, University of Maryland Center for Environmental Science, Baltimore, Maryland, USAa; Delaware Biotechnology Institute, University of Delaware, Newark, Delaware, USAb; Institute for Genome Sciences and Department of Microbiology and Immunology, University of Maryland School of Medicine, Baltimore, Maryland, USAc

## Abstract

Here, we report the draft genome sequence of the estuarine *Synechococcus* sp. strain CB0101. The genomics information of this strain will facilitate the study of the poorly understood *Synechococcus* subcluster 5.2 and how this strain is capable of thriving in a dynamic estuarine system, such as the Chesapeake Bay.

## GENOME ANNOUNCEMENT

The genus *Synechococcus* is a unicellular cyanobacterium that is widely distributed and abundant in the ocean. *Synechococcus* species are members of the picocyanobacteria or picophytoplankton due to their small cell size (1 to 2 µm) and photosynthetic activity. *Synechococcus* spp. can reach 10^6^ cells ml^-1^ and account for a significant portion of phytoplankton biomass and primary production in the Chesapeake Bay during the summer (1, 2). The Chesapeake Bay is subject to various climatic forces that influence the growth and distribution of phytoplankton. Similar to other temperate estuaries, the water temperature ranges from 0 to 30°C in the Chesapeake Bay, and the salinity varies greatly (0 to 30 ppt) from the upper to the lower Bay. Despite the ecological significance of *Synechococcus* in the Chesapeake Bay, little is known regarding how *Synechococcus* spp. survive in such a dynamic ecosystem.

*Synechococcus* sp. strain CB0101 was isolated from the Inner Harbor water of Baltimore, Maryland (39°28′10″N and 76°60′40″W) (3). The isolation of CB0101 led to the discovery of a novel subcluster (subcluster 5.2) within the genus *Synechococcus*, containing members that can thrive in estuarine environments (4). CB0101 is commonly found in the Chesapeake Bay under a wide range of temperatures, salinity, and nutrients (2). Many cyanophages infecting CB0101 have been isolated from the Chesapeake Bay (5). It is believed that *Synechococcus* sp. strain CB0101 is a good model strain to study the adaptation of picocyanobacteria to complex estuarine ecosystems.

The *Synechococcus* sp. strain CB0101 genome was sequenced with a 454 GS-FLX Titanium 8-kb paired-end library. The assembly was performed with Newbler version 2.0.00.22, resulting in 12 scaffolds, 94 contigs, and an N_50_ size of 60,200 bp. Open reading frames were identified and annotated with the J. Craig Venter Institute’s prokaryotic genome annotation pipeline (6). The genome sequence of *Synechococcus* sp. strain CB0101 consists of 2,686,395 bp (64.2% G+C content). The chromosome contains 3,109 genes and 40 tRNA and 2 rRNA gene copies.

The genome revealed that CB0101 has the capacity to sense and respond to changes in its environment. It contains many genes encoding proteins necessary to transport, store, use, and export metals, especially copper, nickel, cobalt, and magnesium. The availability of the genome sequence of CB0101 will allow us to compare it to other model strains in different picocyanobacterial subclusters and to understand the genomic structure of this unique subcluster (7). Members of *Synechococcus* subcluster 5.2 are also dominant in the high-latitude waters (northern Bering Sea and Chukchi Sea), suggesting a possible adaptation of some marine *Synechococcus* spp. in this cluster to cold water. Because marine *Synechococcus* contains multiple clades, it will be interesting to see if the genomic features from different clades are responsible for niche adaptation (to estuarine, coastal, and oceanic environments) or whether, even within specific clades, different strategies have evolved to adapt to these complex environments.

### Nucleotide sequence accession number.

This whole-genome project was deposited in NCBI under the accession no. NZ_ADXL00000000.

## References

[1] Affronti LF Marshall HG 1994 Using frequency of dividing cells in estimating autotrophic picoplankton growth and productivity in the Chesapeake Bay. Hydrobiologia 284:193–203. 10.1007/BF00006689

[2] Wang K Wommack KE Chen F 2011 Abundance and distribution of *Synechococcus* spp. and cyanophages in the Chesapeake Bay. Appl. Environ. Microbiol. 77:7459–7468. 10.1128/AEM.00267-11 21821760 PMC3209163

[3] Chen F Wang K Kan JJ Bachoon DS Lu JR Lau S Campbell L 2004 Phylogenetic diversity of *Synechococcus* in the Chesapeake Bay revealed by ribulose-1, 5-bisphosphate carboxylase-oxygenase (RuBisCO) large subunit gene (*rbcL*) sequences. Aquat. Microb. Ecol. 36:153–164. 10.3354/ame036153

[4] Chen F Wang K Kan J Suzuki MT Wommack KE 2006 Diverse and unique picocyanobacteria in Chesapeake Bay, revealed by 16S-23S rRNA internal transcribed spacer sequences. Appl. Environ. Microbiol. 72:2239–2243. 10.1128/AEM.72.3.2239-2243.2006 16517680 PMC1393199

[5] Wang K Chen F 2008 Prevalence of highly host-specific cyanophages in the estuarine environment. Environ. Microbiol. 10:300–312. 10.1111/j.1462-2920.2007.01452.x 17900294

[6] Tanenbaum DM Goll J Murphy S Kumar P Zafar N Thiagarajan M Madupu R Davidsen T Kagan L Kravitz S Rusch DB Yooseph S 2010 The JCVI standard operating procedure for annotating prokaryotic metagenomic shotgun sequencing data. Stand. Genomic. Sci. 2:229–237. 10.4056/sigs.651139 21304707 PMC3035284

[7] Huang S Wilhelm SW Harvey R Taylor K Jiao N Chen F 2012 Novel lineages of *Prochlorococcus* and *Synechococcus* in the global oceans. ISME J 6:285–297. 10.1038/ismej.2011.106 21955990 PMC3260499

